# Post-transcriptional Regulation of Immunological Responses through Riboclustering

**DOI:** 10.3389/fimmu.2016.00161

**Published:** 2016-04-29

**Authors:** Koelina Ganguly, Jeevan Giddaluru, Avery August, Nooruddin Khan

**Affiliations:** ^1^Department of Biotechnology and Bioinformatics, School of Life Sciences, University of Hyderabad, Hyderabad, India; ^2^Department of Microbiology and Immunology, College of Veterinary Medicine, Cornell University, New York, NY, USA

**Keywords:** inflammation, stress granules, polysomes, mRNA stability, T cell maturation, thymic and peripheral tolerance

## Abstract

Immunological programing of immune cells varies in response to changing environmental signals. This process is facilitated by modifiers that regulate the translational fate of mRNAs encoding various immune mediators, including cytokines and chemokines, which in turn determine the rapid activation, tolerance, and plasticity of the immune system. RNA-binding proteins (RBPs) recruited by the specific sequence elements in mRNA transcripts are one such modifiers. These RBPs form RBP–RNA complexes known as “riboclusters.” These riboclusters serve as RNA sorting machinery, where depending upon the composition of the ribocluster, translation, degradation, or storage of mRNA is controlled. Recent findings suggest that this regulation of mRNA homeostasis is critical for controlling the immune response. Here, we present the current knowledge of the ribocluster-mediated post-transcriptional regulation of immune mediators and highlight recent findings regarding their implications for the pathogenesis of acute or chronic inflammatory diseases.

## Introduction

The transcriptome of an immune cell is subjected to various stages of regulation in the nucleus and cytoplasm before being translated into proteins (1–5). In addition to the basic transcriptional and translational regulation, there exists an intermediate post-transcriptional regulatory (PTR) event, which programs the immunological response according to the changing environmental conditions. This PTR includes the various stages of regulation, including splicing, editing, translation, and decay of mRNAs. Gene transcription and nuclear processes leading to the formation of mature mRNAs in the nucleus are followed by nucleocytoplasmic mRNA trafficking and their exposure to a series of regulatory mechanisms in the cytoplasm. The various functions of dynamic ribonucleoprotein (RNP) complexes in coordinating these nucleocytoplasmic regulatory events have been shown to drive a plethora of immunological responses inside the cells (1–5). The first step in the nuclear post-transcriptional regulatory series is splicing, which involves intron removal and exon joining facilitated by spliceosome complex, formed from the “U” class of small nuclear RNPs (snRNPs). In some cases, splicing also includes a more complex event, the alternative splicing that involves the insertion or excision of specific introns, exons, or regulatory domains in the mature mRNA (6). This is mediated by different classes of RNA-binding proteins (RBPs), including heterogeneous nuclear RNPs (hnRNPs) and serine–arginine RBPs (SR RBPs) (7, 8). The second type of nuclear regulation is editing, which involves the alteration of the nucleotide content of RNA and the subsequent protein translation. The most predominant form of RNA editing in mammals is aided by the adenosine deaminases acting on RNA (ADAR) family of double-stranded RNA-binding enzymes that are responsible for deamination of adenine to inosine (5). Once the functional mature mRNA transcripts exit the nucleus for translation in the cytoplasm, they are exposed to different classes of RBPs based on the mRNA’s cis-regulatory sequences. These RBPs aid in the careful orchestration of mRNA decay in stress granules (SG)/processing bodies (P-bodies) or translation in polysomes, which collectively form an intricate post-transcriptional event (9).

Immune cells can sense and process a wide variety of extracellular and intracellular stress signals *via* cellular stress sensors. Upon activation by cellular stress sensors, the α-subunit of eukaryotic initiation factor 2 (eIF2-α) is phosphorylated, resulting in the attenuation of active translational complex and polysome formation. This results in a pool of translationally stalled uncommitted mRNA transcripts, which then recruit specific RBPs. This recruitment is determined by the cis-acting regulatory elements, such as adenine–uridine-rich elements (AREs) at their 3′-untranslated region (UTR), and forms a RNP complex known as a “ribocluster” (10). The RBPs in the “ribocluster” dictate the location and functionality of mRNAs, determining whether it will be translated or decayed (11). These processes are meticulously coordinated and intricately linked and depend upon the ability of RBPs to interact with the cis-elements, such as AREs at their 3′-UTR of the mRNA transcripts (11, 12). The mRNA transcripts of many immunological mediators, including cytokines and chemokines, have regulatory sequences, such as ARE at 3′-UTR, which allow tight regulation and usage of mRNAs to fine-tune immunological responses as per the cellular requirement (13, 14). The emerging wealth of information regarding RBPs and riboclustering lends credence to its importance in the maintenance of immune homeostasis and programing of the immune response (14). This system has the potential to regulate a wide range of the immune response through the maintenance of equilibrium between synthesis and degradation of the mRNAs that drives immunological reactions, the innate inflammatory responses, immune cell fates, and adaptive host defenses. A better understanding of riboclustering in regulating these integrated pathways might provide leeway toward a development of novel therapeutics. In this review, we present a survey of the current knowledge about riboclustering-mediated post-transcriptional regulation of immune mediators and further highlight recent findings regarding their implications in the pathogenesis of acute or chronic inflammatory diseases.

## Allies in Riboclustering

As introduced in the previous section, riboclusters of spliced mature mRNA transcripts and various RBPs control cytokine and chemokine mRNAs for either subsequent protein expression or exosomal decay (15). The assembly of these riboclusters is largely dependent on the 3′-UTRs (16), the binding of trans-factors like RBPs and non-coding RNAs, such as miRNAs. In this section, we discuss RBPs and cis-elements in more detail.

### RNA-Binding Proteins

RNA-binding proteins, such as tristetraproline (TTP), T cell-restricted antigen 1 (TIA1), TIA1-related protein (TIAR), ZCCHC11, Regnase-1 (also named Zc3h12a, Mcpip1), and ARE-binding degradation factor 1 (AUF1), bind to specific cis-element sequences of cytokine and chemokine mRNAs to form RNP complexes (10). The various RNA-binding domains of different RBPs (14) that have been described so far include RNA recognition motifs (found in TIA1, TIAR, CUGBP2, AUF1, AUF2, and HuR), zinc finger domain (found in BRF1, BRF2, and TTP), and K homology domain [found in fragile X-related protein 1 (FXR1P) and KH-type splicing regulatory protein (KSRP)] (14). Most of the RBPs are found to translocate between the nucleus and cytoplasm (17), and, therefore, riboclustering is hypothesized to dictate the cytoplasmic localization of the bound transcripts and thereby their fate. Various *in vitro* studies have shown that multiple RBPs gain entry onto an RNA transcript, suggesting a cooperative (18) or competitive (19) function by the RBPs to modulate the stabilization or destabilization of a common target transcript.

### Adenine–Uridine-Rich Elements

A few decades ago, clusters of AREs were identified at the 3′-UTRs of newly cloned cytokine mRNAs transcript and were reported to regulate mRNA metabolism (20). The role of ARE in mRNA regulation was quickly confirmed by the decay of heterologous reporter transcripts fused to ARE sequences derived from the 3′-UTRs of granulocyte–monocyte colony-stimulating factor (GM-CSF) mRNA (21). These AREs can act as decisive cis-acting post-transcriptional gene regulatory elements (10). Table 1 provides an interaction profile of the ARE-bearing immunological mediators and proto-oncogenes with specific RBPs. The basic structural components of these AREs are pentamers, nonamers, or clusters of adenine–uridine-rich repeats. These AREs can modulate the cytokine and chemokine levels in cells either independently or by recruiting different groups of RBPs. Table 2 shows the classification of AREs on the basis of sequence information and destabilization kinetics. The information in the table is collected from the database of human ARE-bearing mRNAs created by Bakheet et al. (http://brp.kfshrc.edu.sa/ARED/) (22). This database also suggests that 8% of the mRNA transcribed from human genome bears AREs (22). Much later, it was found that various pro-inflammatory genes, such as those of cytokines, chemokines, and other pro-inflammatory proteins, undergo ARE-mediated decay (AMD) after being subjected to riboclustering (16). However, in the recent years, it has been pointed out that some of these mRNAs are not subjected to decay but rather are stabilized post-transcriptionally when bound to separate class of RBPs (14).

**Table 1:** **Fate of transcripts determined by the interaction with specific RBPs**.

Fate of mRNA	Subjected mRNA	RBP involved in interaction	Reference
Stabilized	IL-3	TTP	(23)
	IL-4	HuR	(24)
	IL-6	TTP and AUF1	(25, 26)
	IL-8	KSRP	(27)
	IL-10	TTP	(28)
	IL-1β	TTP	(29, 30)
	TNFα	TTP, TIA1, FXR1, and HuR	(17, 31–33)
	GM-CSF	TTP	(34, 35)
Destabilized	IL-3	TTP	(10)
	IL-8	TTP, AUF1, and AUF2	(10)
	IL-10	TTP	(10)
	GM-CSF	TTP and AUF1	(10)
	TNFα	TTP, AUF1, AUF2, TIA1, TIAR, and CUGBP1	(10)
	COX2	TTP, AUF1, AUF2, BRF1, and BRF2	(10)
	VEGF	TTP	(10)

**Table 2:** **Classification of ARE sequences on the basis of sequence features**.

Class of ARE	Sequence feature of ARE	Position of ARE	Examples of mRNAs bearing the ARE
Class1	Dispersed pentameric repeats of AUUUA	Within or near a U-rich region in 3′-UTR	c-Myc and c-Fos
Class2	Overlapping non-americ repeats of AUUUAUUUA	Within or near a U-rich region in 3′-UTR	GM-CSF and TNF
Class3	No distinct repeat sequences, U-rich region	3′-UTR	c-Jun

*The information is collected from the database of human ARE-bearing mRNAs created by Bakheet et al. (http://brp.kfshrc.edu.sa/ARED/) (22)*.

## Mechanism of Riboclustering

### Upstream Regulators Involved in the Formation of Riboclusters

The endoplasmic reticulum (ER) is the site for the post-translational modification, folding, and sorting of proteins destined for the secretory pathway of the cell. Calcium disequilibrium or disruption in post-translational modification leads to accumulation of misfolded or unfolded proteins in ER, resulting in ER stress, which can have negative implications on various cellular functions (36). Diseases, such as diabetes, cancer, and neurodegenerative disorders, are often associated with ER stress (37–41). ER stress triggers the activation of transmembrane signaling proteins, including activating transcription factor 6 (ATF6), inositol-requiring protein-1α (IRE1α), and protein kinase RNA (PKR)-like ER kinase (PERK). This pathway helps to establish ER homeostasis by increasing chaperone expression, sequestering mRNAs from polysomes, and ER-assisted degradation (ERAD) of the misfolded proteins in the cytosol (42). During amino acid depletion, the intracellular sensor general control non-derepressible 2 (GCN2) gets activated by the accumulation of uncharged tRNAs, while HRI senses heme deprivation within the cell. As a part of the antiviral response, PKR gets activated by viral dsRNA, interferons, and growth factors (43). The four vital sensors of stress pathway, namely GCN2, PERK, PKR, and HRI, undergo activation by phosphorylation in response to the above-mentioned stress conditions. Phosphorylated sensor molecules, in turn, phosphorylate eIF2-α (44) and prevent the recruitment of eIF2–GTP–methionyl initiator tRNA onto the 40S ribosomal subunit. This, in turn, leads to the translation initiation failure owing to stalled formation of 43S preinitiation complex and its subsequent binding to eI4F, and other factors required for the assembly of the active polysomes (44). Alternatively, nutrient deprivation or infection activates TSC proteins that negatively regulate the metabolic checkpoint control molecule mammalian target of rapamycin complex 1 (mTORC1). Upon deactivation of mTOR, eIF4E-binding proteins (eIF4E BP1 and BP2) are capable of inhibiting the formation of the preinitiation complex by preventing the interaction of eIF4F complex to the 5′ cap of mRNA (45). Thus, when cells are exposed to oxidative damage, infection, or nutrient deprivation, there is a transient decrease in global protein synthesis as a mode of adaptation to environmental stresses. This results in a pool of translationally stalled mRNAs and disintegrated translation machinery. These uncommitted mRNA transcripts then recruit specific RBPs, determined by the sequence elements in the regulatory domains, to form “riboclusters” (10).

The composition of riboclusters is in constant flux, with some RBPs gaining or losing access during the mRNA’s journey from transcription to translation or decay. The RBPs in the ribocluster dictate the location and functionality of mRNAs as to whether it will be subjected to translation in polysomes, saved in a cache in SGs for further use, or otherwise decayed in P-bodies (11). All of these processes are highly coordinated and intricately linked and depend upon the ability of RBPs to interact with the regulatory sequences or structures present in the UTRs of the mRNA transcripts (11, 12). The presence of such regulatory sequences or elements in the mRNA transcripts encoding many immunological mediators, including cytokines and chemokines (13, 14), allows coordinated regulation and usage to tune immunological responses “on” or “off” as per the cellular requirement.

### The Downstream Process

**Figure 1:**
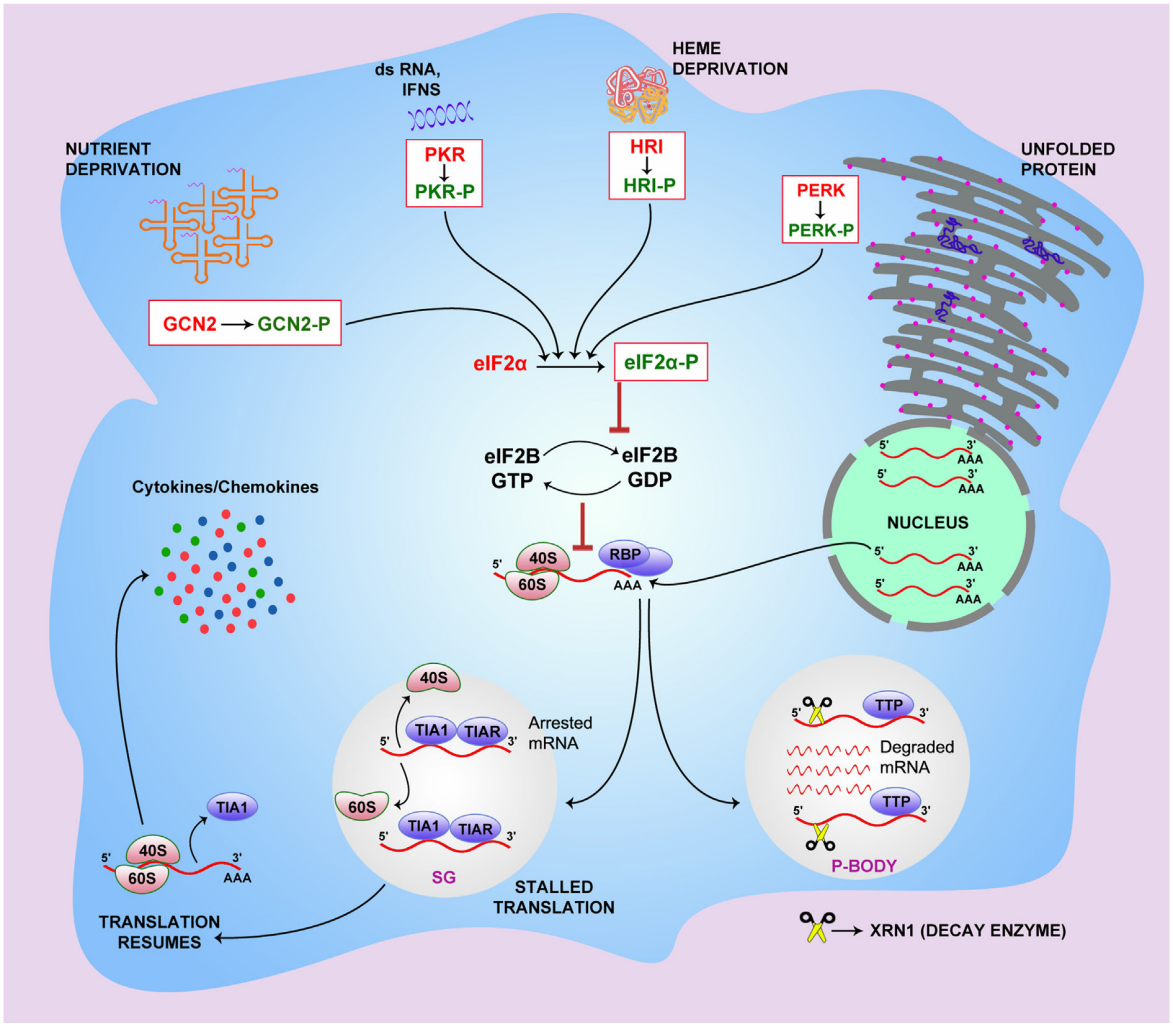
**The link between integrated stress pathway and riboclustering**. General control non-derepressible 2 (GCN2), PKR-like endoplasmic reticulum kinase (PERK), protein kinase RNA (PKR), and heme-regulated initiation factor 2-alpha kinase (HRI) stress sensors get activated when the cell is subjected to specific stress conditions. PERK senses endoplasmic reticulum stress caused by misfolded or unfolded proteins, GCN2 senses accumulation of uncharged tRNAs under conditions of amino acid unavailability, and the HRI molecule senses heme deprivation within the cell. PKR gets activated by viral dsRNA, interferons, and growth factors. Upon activation, these sensor molecules undergo phosphorylation, which subsequently phosphorylate eukaryotic initiation factor 2-α (eIF2-α), preventing the recruitment of eIF2–GTP–methionyl initiator tRNA onto the 40S ribosomal subunit. This, in turn, leads to the translation initiation failure owing to retarded GTP–GDP exchange and stalled formation of 43S preinitiation complex. In cells exposed to stress, RBPs, including T cell-restricted antigen 1 (TIA1) and TIA1-related protein (TIAR), bind to stalled mRNAs and propel preinitiation complexes of translation to stress granules (SGs) for temporary silencing. The sorted mRNA transcripts, destined for translational arrest, are delivered to the processing bodies (P-bodies) for degradation using decay enzymes like 5′–3′ mRNA decay enzyme Xrn1, decapping enzyme, and others. RBP-mediated export of the mRNAs in the SGs from polysomes is reversible. Higher concentrations of stabilizing factors, such as HuR, ensure rescue of the transcripts from SGs leading to translation initiation, while increased recruitment of destabilizing factors, such as TTP, BRF1, and BRF2, leads to SG formation and subsequent mRNA decay in P-bodies.

In cells that are exposed to stress, various RBPs, such as TIA1 and TIAR, interact with stalled mRNAs and propel translation preinitiation complexes to discrete cytoplasmic foci of SGs and P-bodies (46). P-bodies bear all the decay enzymes, including 5′–3′ mRNA decay enzyme Xrn1, decapping enzyme, and others (11). The sorted mRNA transcripts destined for translational arrest are delivered from the SGs to the P-bodies either for degradation or temporary silencing. However, the RBP-mediated export of the mRNAs in the SGs from polysomes is reversible in nature (12). Higher concentrations of stabilizing factors, such as HuR, ensure the rescue of the transcripts from SGs and result in translation initiation, while increased recruitment of destabilizing factors, such as TTP, BRF1, BRF2, and others, leads to SG formation and subsequent mRNA decay (13, 47). TIA1 has been found to coprecipitate with KSRP from the TNFα ARE (14), which may explain the temporal sequence of events involved in post-transcriptional regulation of the TNFα mRNAs. After being bound to TIA1/TIAR, TNFα mRNAs are removed from polysomes and are targeted for decay within the P-bodies in association with KSRP (48). Figure 1 shows the current understanding of the mechanism that connects the stress pathway and post-transcriptional regulation of immunological mediators, including cytokines and chemokines.

## Riboclustering in Innate Immunity: Initiation, Perpetuation, and Resolution of Inflammation

The immune system has to respond to invading pathogens by generating a pro-inflammatory response, which eventually has to be suppressed, allowing a return to homeostasis. In response to such changing immune microenvironments, riboclusters change their composition and cytosolic locations in the responding immune cells, and thereby play essential roles in driving the initiation through the resolution phase of inflammation by a prudent balancing of the pro-inflammatory and anti-inflammatory cytokines and chemokines. Pathogen products, such as lipopolysaccharides (LPS), dsRNA, and flagellin, are recognized by pathogen-associated molecular patterns (PAMPs) or PAMP-recognizing receptors (PRRs). Common PRRs, such as toll-like receptors (TLRs) and NOD-like receptors (NLRs) (49–51), are present on/in immune cells, such as antigen-presenting cells (APCs). These receptors subsequently initiate a set of signaling cascades culminating in the activation of different transcription factors, such as nuclear factor kappa B (NF-κB), activator protein-1 (AP-1), interferon regulatory factors (IRFs), and CCAAT/enhancer-binding protein β (C/EBPβ) (52). The activation of different transcription factors is marked by an elevation in mRNA transcripts encoding for pro-inflammatory cytokines, such as IL-1β, IL-6, and TNFα (52).

Activation of these PRRs also triggers riboclustering *via* the p38-mitogen-activated protein kinase (MAPK) pathway, which phosphorylates TTP at specific serine residues (Ser 52,178) (53). Phosphorylation of TTP reduces its binding to cytokine mRNAs, allowing these mRNAs to be translated. The reversion of TTP from its inactive phosphorylated state to its active (i.e., mRNA-destabilizing) dephosphorylated state is prevented by the recruitment of the 14-3-3 protein, which blocks the interaction of TTP with decay enzymes and phosphatase 2α, thus retaining its phosphorylated state (54). Alternative to the p38-MAPK signaling pathway, several other pathways have been identified in the regulation of cytokine expression, such as TNFα. Initiation factor eIF4G-mediated recruitment of Mnk1 (MAPK signal-integrating kinases) onto the 5′ cap-binding protein eIF4E results in phosphorylation and inactivation of translation silencer hnRNPA1, which regulates TNFα expression. This is manifested in the release of TNFα mRNA from hnRNPA1, followed by cap-dependent translation initiation of TNFα upon triggering of this pathway (55–57).

**Figure 2:**
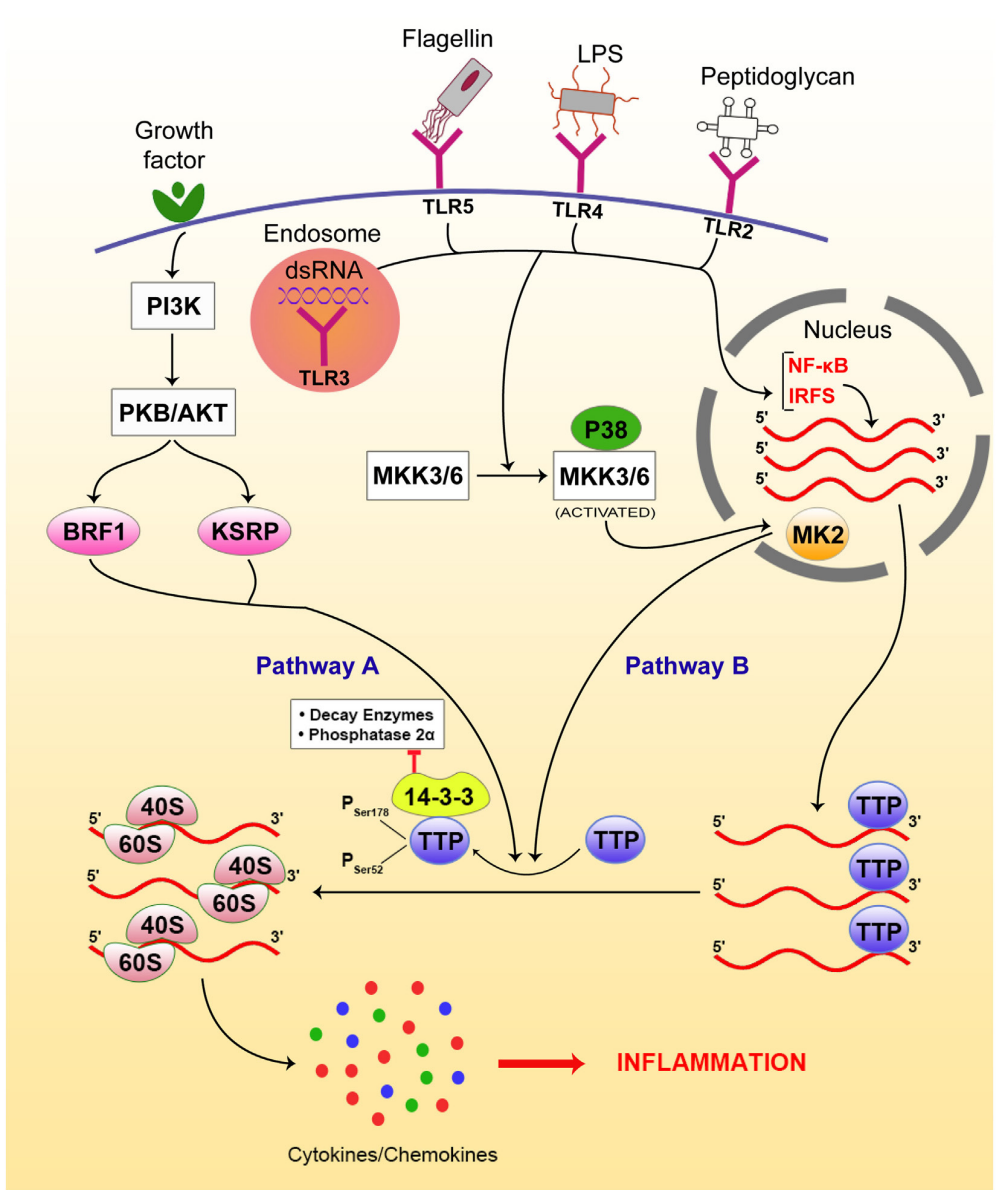
**Riboclustering-mediated signaling pathways that regulate innate inflammatory modulators, including cytokines/chemokines**. There are two dominant pathways that mediate the process. Toll-like receptors (TLRs) bind to specific ligands, get activated, and induce p38-mitogen-activated protein kinase (MAPK) pathway *via* nuclear factor (NF)-κB pathway signaling intermediates. On the other hand, the growth factor receptors activate phosphatidylinositol 3-kinase (PI3K), activating protein kinase B (PKB/AKT), which phosphorylates BRF and KSRP. Both pathways contribute to post-transcriptional regulation of cytokine/chemokine mRNAs. Upon phosphorylation by either of the above pathways, TTP gets inactivated and looses access from the 3′-UTRs of the mRNA transcripts, freeing them from SGs and allowing their translation, resulting in inflammation.

In an analogous pathway, signals from growth factor activate the PI3-kinase pathway, which then activates protein kinase B/AKT, which in turn phosphorylates the secondary modifiers BRF1 and KSRP. Phosphorylated BRF1 and KSRP phosphorylate TTP, as in the case of the p38-MAPK pathway. Upon phosphorylation, the TTP loses its ability to bind to cytokine mRNA, such as IL-3, thereby resulting in the stabilization and translation of IL-3, a major cytokine in hematopoiesis (58, 59). Figure 2 gives an overview of the signaling pathways involved in this process.

These pathways control the cytokines involved in the resolution of inflammation and maintenance of tissue homeostasis mainly through the production of anti-inflammatory mediators, such as IL-4, IL-10, and TGFβ (60). The decay of the pro-inflammatory cytokine mRNAs is another essential determinant of inflammatory inactivation (60) and is promoted by the dephosphorylation of either the destabilization factor, TTP (53, 61), or that of the secondary modifiers BRF1 and KSRP. Once dephosphorylated, they tether to the subjected mRNAs leading to their sequestration to the SGs or P-bodies from the cytosol.

The importance of TTP is illustrated by the fact that TTP knockout mice are highly susceptible to severe inflammatory challenges marked by chronic lung and joint pathology, dermatitis, cachexia, and myeloid hyperplasia (60). However, these conditions can be ameliorated by blocking pro-inflammatory mediators TNFα, CCL3, IL-23, and IL-17 (62–66). Translational silencers TIA1 and TIAR stall formation of translation preinitiation complexes and propel the arrested pro-inflammatory mRNAs from the polysomes to the SGs or P-bodies for degradation (9). Thus, deletion of TIA1 results in aggravation of syndromes in TTP-deficient mice (67). Furthermore, this pathway of mRNA homeostasis can be controlled by the anti-inflammatory cytokines IL-4, IL-10, and TGFβ, which promote TTP expression (68–70).

Besides TTP, dephosphorylation of Regnase-1 protein is also involved in the destabilization of inflammatory cytokine mRNA, such as IL-6. Upon TLR activation, Regnase-1 gets phosphorylated and deactivated in a similar manner to TTP. While in resting condition or anti-inflammatory state, dephosphorylation results in the activation of Regnase-1, allowing it to gain access to the 3′-UTR of IL-6 mRNA and subject the latter to decay (71). Similarly, AUF1 is reported to form dimers and bind to AREs of pro-inflammatory mRNAs (72). Knockout studies show that AUF1-deficit mice are susceptible to acute and chronic inflammatory dermatitis because of high levels of pro-inflammatory cytokines. This clearly indicates that AUF1 is a crucial regulator of the immune response that attenuates the translation of selective pro-inflammatory cytokine transcripts (29, 73).

Other destabilizing factors, such as Roquin proteins 1 and 2, not only target the ARE sequences of the mRNAs but also bind to the constitutive decay elements (CDEs) of TNFα mRNA resulting in its exonucleolytic decay as mediated by TTP in ARE-mediated (AMD) (74). Indeed, knockout experiments suggest that mice lacking Roquin proteins are highly susceptible to TNFα-mediated inflammatory syndromes (75, 76).

Viral ssRNA and dsRNA are detected by the intracellular receptors TLR 7/8 and TLR 3/9, retinoic acid-inducible gene-I (RIG-I), melanoma-differentiation-associated gene 5 (Mda-5), and DHX33 [DEAH (Asp–Glu–Ala–His) Box Polypeptide 33], respectively (49, 77, 78). This interaction results in the production of interferons, which initiate several antiviral processes inside the cell. Interferon activates PKR, which triggers shut down in host translation through blockage of eIF2 (79). It also induces ADAR enzymes to control the translation or decay of the existing mRNAs by ARE-mediated post-transcriptional regulation (5).

Certain RBPs can both repress and de-repress inflammatory mechanisms, depending on the RNP configurations with which they are associated at a certain point of time. For example, the RBP HuR was initially thought to be a stabilizing RBP in the context of ARE-bearing mRNAs. However, mice lacking HuR are more prone to hypersensitive immune disorders due to increased levels of ARE-bearing pro-inflammatory molecules, which support a destabilizing role of HuR (18, 80). However, HuR can play both pro-inflammatory as well as anti-inflammatory roles (80), since HuR and TIA1 cooperatively inhibit translation of pro-inflammatory cytokines, such as IL-1β and TNFα (18). On the other hand, HuR has been found to inhibit AMD by TTP and AUF1, while itself acting synergistically with TIA1 in promoting such decay process (18).

Additionally, other translational blockades are activated inside the cell in cooperation with RBPs that drive the anti-inflammatory processes. When pro-inflammatory modulators reach a critical concentration; they drive autocrine and paracrine regulatory pathways. For instance, IFNγ directs diversion of ribosomal protein L13a onto GAIT (IFNγ-activated inhibitor of translation) elements of mRNA transcripts, such as CCL22, CXCL13, and CCL8, and curbs their translation (81–83). Moreover, the equilibrium between the inflammation and tissue homeostasis is maintained in part by the serine/threonine protein kinase B (Akt)/mTOR pathway (84). For example, during infection with virulent *L. pneumophila* (85), mTOR-activating kinase Akt gets degraded, and TSC proteins are released, culminating in the inhibition of mTORC1-mediated translation while pro-inflammatory cytokines are readily translated (85). By contrast, during infection with avirulent *L. pneumophila*, mTOR complex 1 (mTORC1) is activated by loss of TSC proteins, resulting in increased synthesis of immunoregulatory molecules and decay of mRNAs encoding for pro-inflammatory factors (86). The ability of mTOR to regulate translation inhibition and elevated expression of pro-inflammatory cytokines may be due to ARE-BPs. Indeed, metabolic alterations that activate AMP-activated protein kinase can inhibit mTOR-related translation and destabilize RBPs (87, 88). Furthermore, the inactivated mTOR was still able to bind to the destabilizing RBP TIAR, thus leaving the 3′ARE sequences in the mRNA unoccupied (89). This renders the cytokine mRNAs to be ready for translation and could explain their higher concentrations in the absence of active TOR in the cell. Thus, the interactions with different ARE-BPs may be crucial in this mTOR-mediated counterbalance between perpetuation and repression of inflammation.

## Riboclustering and Other Post-transcriptional Modifications in Adaptive Immunity

Alongside the innate responses, the adaptive arm of the immune system plays a crucial role in the immune response. RBP-mediated riboclustering and other PTRs have been noted to be critical for maturation, selection, activation, and tolerance of adaptive immune cells. Although its role in B cell biology is not well studied, its role in the genesis and generation of T cell responses has been well-described. Below, we focus on some of these aspects.

### T Cell Maturation and Thymic Tolerance

In the thymus, progenitor T cells develop into naive T cells *via* sequential developmental stages from CD4^−^/CD8^−^ double negative (DN) T cells to CD4^+^/CD8^+^ double positive (DP) T cells, and finally to lineage-specific naive T cells with rearranged T cell receptor (TCR) αβ or γδ receptors. Each of these events is controlled in part by RNP complexes regulated by Notch–Delta and pre-TCR signaling. In developing thymocytes in the thymic cortex, Notch mRNA is controlled by AMD imposed by two TTP variants, ZFP36L1 and ZFP36L2 (90). Before the replacement of Notch receptor by a pre-TCR receptor, ZFP36L1 and ZFP36L2 are subjected to Notch signaling induced phosphorylation (59). During the rearrangements of pre-TCR β receptor mRNAs, the upstream frame shift proteins (Upf1 and 2) are involved in removing the unfolded or misfolded variants (91, 92). Ribosomal protein S6, a major substrate of mTOR, also plays a major role in this process as S6-deficient mice exhibit blocked transition of DN to DP thymocytes due in part of the inability to counteract p53-mediated genotoxic shock resulting from the lack of functional ribosomes (93). The successful transcription of the TCRα gene in DP cells is critical for the development of T cells, allowing expression of the complete receptor for lineage committed naive T cells and allowing them to leave the thymus to participate in the immune response in the periphery. TCRα gene transcription is attenuated by the splicing functions of CUGBP family of RBPs (94). Upon induction by pre-TCR signaling, the CUGBP family protein CUGBP Elav-like family member 2 (CELF2) cause rearrangements in the lymphoid enhancer-binding factor 1 (LEF1) transcription factor mRNA, allowing LEF1 to bind to the enhancer region of the gene encoding TCRα in the DP cells, leading to the development of the final mature TCR αβ complex (94).

**Figure 3:**
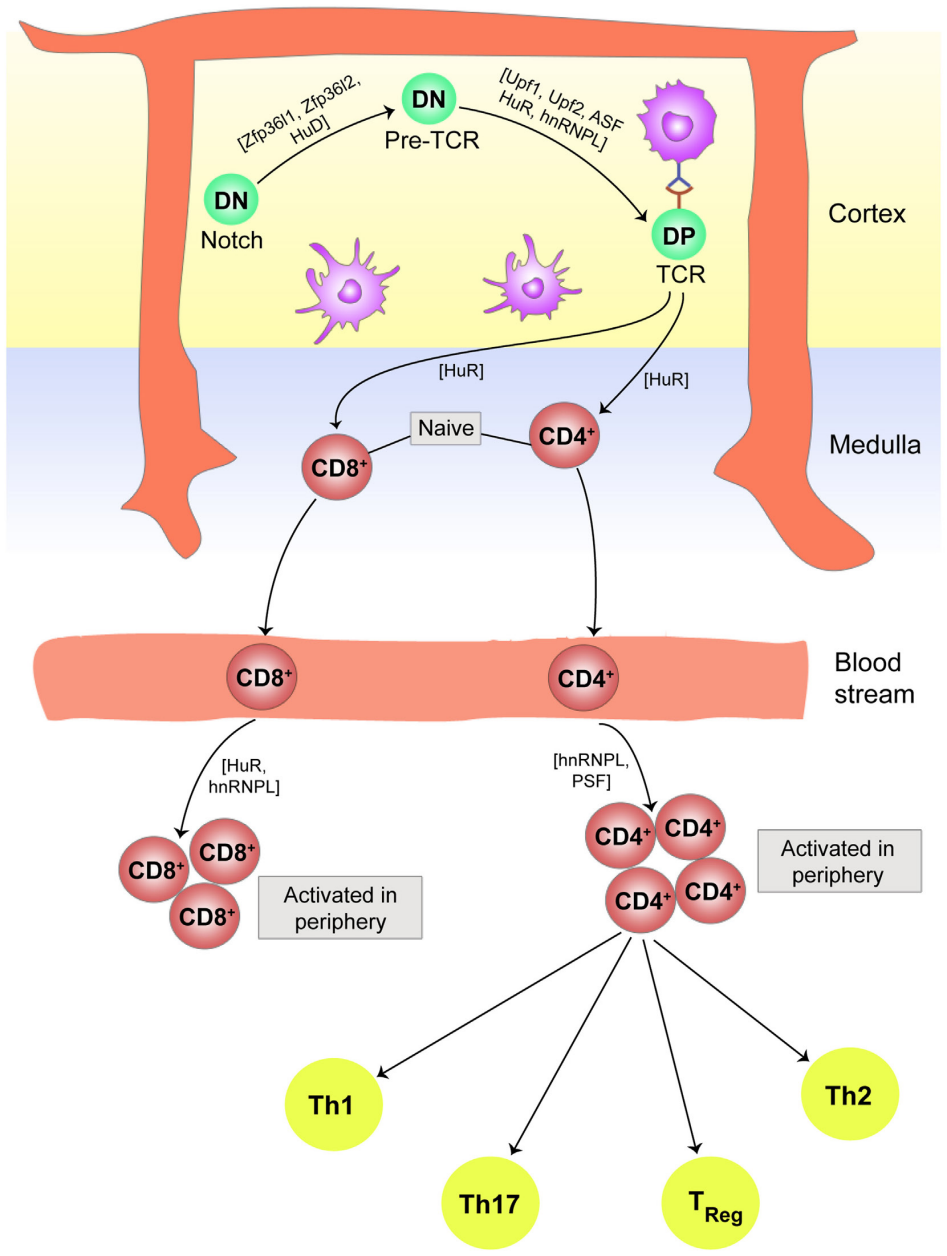
**Post-transcriptional regulation plays an essential role in T cell development, maturation, and commitment**. In the thymic cortex, double negative (DN) thymocytes experience Notch signaling followed by development of pre-T cell receptors (TCR) and finally end up as double positive (DP) T cells with fully processed TCR. The entire path traversed by a naive T cell to single positive (SP) CD4 or CD8 T cell, to its movement into the peripheral blood stream for activation after antigen, is controlled in part by ribonucleoprotein complexes. In the periphery, activated CD4^+^ T cells differentiate into subsets of Th17, Th1, Th2, T regulatory cells, and other effector T cells dependent on the cytokine environment in the vicinity. Cytokines and chemokines are in turn modulated by riboclustering, depending upon the nature of the invading pathogen.

T cells with mature TCR also bear CD3 receptor complexes, which transduce TCR signals from antigen-bound major histocompatibility complex (MHC). The strength of TCR–MHC interaction along with co-stimulation from the other accessory surface proteins guides the selection of non-self-reactive CD4^+^ or CD8^+^ cells (positive selection), or elimination of self-reactive CD4^+^ or CD8^+^ cells (negative selection) in the thymus. These intricately orchestrated selection processes are regulated at multiple stages by RBPs. The CD3ζ chain is the major signaling subunit of the CD3 receptor complex of the TCR. Proper splicing of CD3ζ pre-mRNA is essential for effective TCR signaling, since improperly spliced CD3ζ mRNA lacking either a part of the coding region or a part of their 3′-UTR cannot recruit HuR, and the mRNA becomes prone to degradation. In the absence of functional CD3ζ, the TCRαβ cannot deliver the proper signal required for T cell thymic selection, resulting in the removal of the T cell (95). CD3 chains also interact with ZAP 70, a TCR-associated kinase, which is phosphorylated by Lck, a receptor-proximal Src kinase. Lck is in turn activated by transmembrane tyrosine phosphatase CD45, which dephosphorylates the C-terminal inhibitory tyrosine residue (Tyr505) on Lck (96). CD45 exists in various alternatively spliced isoforms with different functions. The shorter isoform CD45RO is the less active isoform on the surface of immature T cells, while the longer one is the fully functional variant CD45RB and is expressed on the mature CD4 and CD8 T cells. Serine/arginine-rich RBPs, arginine/serine splicing factor (ASF) and SRSF2, and the heterogeneous nuclear RNP hnRNPL are primarily responsible for alternative splicing of the CD45 pre-mRNA (97–99). The knockout experiments have identified that RBP HuR plays crucial role in TCR signaling by differential targeting of the mRNAs with AREs (100). The HuR-deficit cells and hnRNPL-deficient cells show similar phenotypes in the migration of mature T cells from thymic cortex to the medulla and finally into the peripheral circulation (99, 101), suggesting that this pathway also regulates the export of T cell from the thymus to the periphery. Figure 3 gives a brief overview of the role of PTRs in T cell development, maturation, and commitment in the thymus and periphery, respectively.

### T Cell Anergy and Peripheral Tolerance

Once the T cells mature and leave the thymus, they can recognize the antigen in the secondary lymphoid organs. There are typically three sets of signals for successful T cell activation and clonal expansion. The first signal comes from the binding of the TCR to its cognate peptide-bound MHC on the APCs. The second signal is provided by co-stimulation from the CD28–B7 interaction. The third signal is imparted by the cytokines secreted by immune cells in the vicinity. The different isoforms of CD45 are expressed by naive, activated, or memory T cells. Naive T cells express a longer isoform of CD45 (CD45RA) while activated, and memory T cells express the spliced shorter form (CD45RO). This stage-restricted splicing phenomenon of CD45 is aided by hnRNPLL, a paralog of hnRNPL and PSF or polypyrimidine tract-binding protein (PTB)-associated splicing factor (102, 103). A genetic mutation in hnRNPLL results in defective binding to CD45 mRNA, and improper alternate splicing result in decreased number of memory T cells (103).

The co-stimulatory signals from the CD28 superfamily prevent T cell anergy and allow the activated T helper cells to differentiate. The T cell co-stimulatory molecules OX-40 and ICOS are under post-transcriptional regulation by the decay factors Roquin-1, Roquin-2, and Regnase-1 (104). The deletion of these factors either individually or in combination interfere with the T cell co-stimulation (76, 105). Furthermore, the miRNA-mediated silencing of Roquin proteins leads to the activation of CD28, OX-40, and integrin LFA1 in CD4^+^ T cells (105). This subsequently activates the PIN1 prolyl isomerase, which targets the phosphorylated RBPs to form proline isomers (106). This alteration in the conformation of the RBPs reduces their affinity for the ARE sites of translationally arrested transcripts. A similar level of control exists during T cell activation as a result of CD40 and CD40L interaction, with cytoplasmic stabilization of CD40L pre-mRNA being under the control of nuclear RNPs hnRNPL and nucleolin (107). By contrast, in the absence of CD28 co-stimulation resulting in T cell anergy, mRNAs accumulate, ultimately leading to translational inhibition. This is controlled by the mTORC1 pathway (108).

Cytokine signaling imparts a tertiary function that is required for T cell activation, clonal expansion, and downstream effector functions. The IL-2 functions in autocrine signaling for the T helper cell proliferation. The 3′-UTR of IL-2 mRNA can recruit destabilizing factors TTP, Regnase-1, and the nuclear transcription factor NF90 (109). Moreover, in primed CD4^+^ T helper cells, the IL-4 mRNA is sequestered in the SGs by the aid of TIA1 until a second exposure to the antigenic signal (110). The HuR can also stabilize the mRNA for other cytokines, such as IL-17, which was recently found to be responsible for autoimmune neuroinflammation (111).

Once the mature T cells are out into the periphery, they undergo another round of scrutiny, distinctively called peripheral tolerance. Self-reactive T cells undergo elimination by apoptosis or programed cell death. This is controlled in part by the cell surface protein Fas/CD95. The Fas/CD95 undergoes alternative splicing, giving rise to two isoforms, a membrane-bound form that facilitates apoptosis and a soluble form that opposes the same. HuR, hnRNP C1, and PTB favor the inclusion of the sixth exon in Fas mRNA, while TIA1, TIAR, and hnRNPA1 block this process (112). The evidence for the regulation of this process by RBPs include that fact that HuR-deficit T cells are resistant to apoptosis (101, 113), and Fas splicing is arrested when U2 snRNPs are cleaved by effector caspases (114). Given the role of Fas/FasL in autoimmune disorders, this suggests a link between PTRs and autoimmune disorders.

## Conclusion and Future Prospects

Immune cells exhibit a wide array of temporally and spatially regulated functions in response to a plethora of intracellular and extracellular signals. For example, during inflammation, cytokine mRNAs are induced and gradually subside when the causal agent is removed from the system leading to tissue homeostasis and regeneration. This prompt response of the host cell to the invader and subsequent return to homeostasis suggest the involvement of PTRs, including riboclustering. The mRNA transcripts encoding some of the major effector molecules of this immune response are either kept in cache until they are translated to manifest its effector machinery depending on the needs of the cell or are subjected to alternative splicing, leading to the formation of various isoforms of T or B cell surface molecules. There is strong evidence that riboclustering plays a critical role in this process, and can, therefore, be said to be a key immune-sorting mechanism for these cells. The PTR events are carefully orchestrated for precise coordination of regulatory actions of RBPs and non-coding RNAs, on coding RNA transcripts. Future research should focus on developing approaches to identify these specific RNA targets for individual RBPs, and the cumulative roles of these RNP complexes in response to various signals. To date, various approaches used to understand these functions include overexpression, knockdown, or knockout experiments. However, these approaches come with significant caveats including the fact that a point or domain mutation in RBPs, complete shutdown, or overexpression in the cell may result in stress-related alterations that may be misleading in extrapolating the function of the RBP. Thus, system biological approaches or other experimental approaches promise to reveal more about the crucial regulatory system in the cell. Macromolecular tracer techniques and live cell imaging can also be beneficial in this respect. The future research on riboclustering and elucidation of the molecular pathways involved may usher novel therapeutic approaches and decipher probable drug targets for inflammatory diseases, autoimmune disorders, and cancer.

## Author Contributions

KG wrote the paper, JG made the figures, AA critically revised the manuscript, and NK contributed to both writing and critical revision of the paper.

## Conflict of Interest Statement

The authors declare that the research was conducted in the absence of any commercial or financial relationships that could be construed as a potential conflict of interest.
